# Individual and Community-Level Factors Associated With Intention to Use Contraceptives Among Reproductive Age Women in Sub-Saharan Africa

**DOI:** 10.3389/ijph.2022.1604905

**Published:** 2022-06-29

**Authors:** Desale Bihonegn Asmamaw, Habitu Birhan Eshetu, Wubshet Debebe Negash

**Affiliations:** ^1^ Department of Reproductive Health, Institute of Public Health, College of Medicine and Health Sciences, University of Gondar, Gondar, Ethiopia; ^2^ Department of Health Education and Behavioral Sciences, Institute of Public Health, College of Medicine and Health Sciences, University of Gondar, Gondar, Ethiopia; ^3^ Department of Health Systems and Policy, Institute of Public Health, College of Medicine and Health Sciences, University of Gondar, Gondar, Ethiopia

**Keywords:** family planning, intention to use, married women, socio-economic status, sub-Saharan Africa

## Abstract

**Objectives:** The present study identifies factors that affect intention to use contraceptives among married women in countries in sub-Saharan Africa.

**Methods:** Secondary data analysis was conducted using Demographic and Health Surveys. A total of 334386 weighted sample women who were fecund were included in the analysis. Multi-level mixed-effect logistic regression analysis was fitted to identify individual and community level factors associated with intention to use contraceptives.

**Results:** The prevalence of intention to use contraceptives was 45.76%. Age, educational status, and wealth quintile were factors affecting the intention to use contraceptives.

**Conclusion:** Several individual and community level factors were associated with the intention to use contraceptives in SSA. Therefore, governmental and non-governmental organizations should consider these factors when implementing strategies.

## Introduction

The intention of using the contraceptive method is imperative to understanding a woman’s future needs and improving her chances of converting those intentions into action [1]. It is widely believed that intentions predict behavior and that in many behavior change interventions, including those targeting contraceptive use, behavioral intentions are used to evaluate program effectiveness [2]. Moreover, the intention is used as an indicator of potential demand for family planning services [3].

Women’s access to life-saving contraception should be recognized as a human right [4]. A person’s intention to perform a behavior is driven by perceived costs and motivation [5]. Intention to use and use of family planning has been the best method to control family size and unnecessary pregnancies [6]. Even the intention to use contraceptives improves the health of children, women, families, and whole societies [7]. In sub-Saharan Africa, unintended pregnancies, high fertility, and abortion are still challenges for women of reproductive age [8].

As the number of children rises to four or more, there will be an increase in the risk of maternal mortality [9]. To regulate fertility, implementing a family planning strategy is very important [10]. In the year 2020, the London summit aimed to mobilize service deliveries to an additional 120 million mothers in the world’s poorest countries to use contraceptives [11]. Achieving this objective could prevent 100 million unintended pregnancies, 50 million abortions, 3 million infant deaths, and 21 thousand maternal deaths [12]. To achieve the Sustainable Development Goal (SDG3), which calls for universal access to sexual and reproductive health services by 2030, ensuring universal access to sexual and reproductive healthcare services is mandatory [13]. But developing countries, particularly Sub-Saharan Africa, is still under low utilization of contraceptives [14].

In African countries, women’s intention to use contraception has been influenced by their partners’ fertility preference [15], parity [15], desire for children [16], misconceptions about contraceptives [17], and sociodemographic factors like marital status, residence, already having children, age, and religion [15, 18]. Through considering these factors, assessing the intention to use contraception brings tangible evidence for intervention in the sub-Saharan African countries [19].

Most of the previous studies in sub-Saharan Africa on intention to use contraceptives were institutional based and restricted to specific countries, regions, or zones and with a small sample size. However, this study seeks to use nationally representative data; we can better understand individual and community level determinants of intention to use contraceptives. The study is important because the result will be useful as an input for program planners and resource allocators to predict future behaviors and to be effective in their program implementation. Additionally, the study helps to develop an effective behavior change communication strategy and is an insight to demand and future use of contraceptives.

## Methods

Data for this study were obtained from the most recent Demographic and Health Surveys (DHS) of 33 SSA countries, which were conducted between 2010 and 2019. The DHS is a nationally representative household survey that is conducted across low- and middle-income countries every 5 years [20]. We used the women’s recode (IR file) data set and extracted the dependent and independent variables. The dataset is freely available for download at: https://dhsprogram.com/data/available-datasets.cfm.

The DHS employs a two-stage stratified sampling technique. This makes the data nationally representative. A total weighted sample of 334386 married reproductive age women were included in the study. Women with infecundity were excluded. Details about the selected countries, the year of the survey, and the sample are shown in Table 1.

**TABLE 1:** Sample size for intention to use contraceptives in countries in sub-Saharan Africa for each country, 2010–2019.

Regions	Country	Year of survey	Weighted sample (n)	Weighted sample (%)
East Africa countries	Burundi	2016/17	14023	4.19
	Comoros	2012	4571	1.37
	Ethiopia	2016	11559	3.46
	Kenya	2014	8201	2.45
	Malawi	2015/16	13017	3.89
	Mozambique	2011	11926	3.57
	Rwanda	2014/15	8926	2.67
	Tanzania	2015/16	8935	2.67
	Uganda	2016	12743	3.81
	Zambia	2018	8683	2.60
	Zimbabwe	2015	5069	1.52
Central Africa countries	Angola	2015/16	12443	3.72
	Cameroon	2011	10896	3.26
	Chad	2014/15	16087	4.81
	The Democratic Republic of the Congo	2013/14	14886	4.45
	Republic of the Congo	2011/12	5953	1.78
	Gabon	2012	5551	1.66
West Africa countries	Benin	2017/18	13571	4.06
	Burkina Faso	2010	14367	4.30
	Ivory Coast	2011/12	7874	2.35
	Gambia	2013	10246	3.06
	Ghana	2014	7163	2.14
	Guinea	2018	9545	2.85
	Liberia	2013	5906	1.77
	Mali	2018	8763	2.62
	Niger	2012	9707	2.90
	Nigeria	2018	35545	10.63
	Senegal	2010/11	7012	2.10
	Sierra Leone	2013	11694	3.50
	Togo	2013/14	7472	2.23
Southern Africa countries	Lesotho	2014	3301	0.99
	Namibia	2013	4448	1.33
	South Africa	2016	4302	1.29
Total sample size			334386	100

The outcome variable for this study was intention to use contraceptives among married reproductive age women. We measured the variable as use later (1 = yes) and unsure about use or not intend (0 = no). We incorporated several individual and community level independent variables based on available evidence on the intention to use contraceptives among reproductive age women. Media exposure (yes, no) was coded as yes if the women heard the message either in the newspaper, radio, or television at least once a week, and no for otherwise. Wealth index was constructed by household asset data via Principal Component Analysis (PCV) to categorize individuals into wealth quintile (poor, middle, and rich).

In DHS, except for residence and country, all the other factors were collected at the individual level. Hence, we generated three community-level factors, namely community level women’s education, community-level poverty, and community-level media exposure, by aggregating the individual-level factors at cluster level and categorizing them as high and low based on the median value.

Stata version 14 statistical software was used for data analysis. All frequency distributions were weighted (v005/1000000) throughout the analysis to ensure that the DHS sample was a representative sample and to obtain reliable estimates and standard errors before data analysis.

The first step was a graphical representation of the intention to use contraceptives among reproductive age women in SSA. The second step was a bivariate analysis that calculated the proportion of intention to use contraceptives across the independent variables with their *p*-values. All the variables that were statistically significant in the bivariable analysis were used for multi-level analysis. In the final step of the analysis, a multilevel logistic regression analysis comprising fixed effects and random effects was done.

The results of the fixed effects of the model were presented as adjusted odds ratio (AOR) while the random effects were assessed with Intra-Cluster Correlation (ICC). Four models were fitted: null model (Model 0) which shows the variations in the intention to use contraceptives in the absence of any independent variables; Model I, which adjusted for the individual-level variables; Model II, which adjusted for the community level variables; and Model III, which adjusted for both individual and community level variables. Simultaneously, Model fitness was done using the deviance (-2LLR).

Results were presented as adjusted odds ratios (AOR) at 95% Confidence Interval.

## Results

**FIGURE 1:**
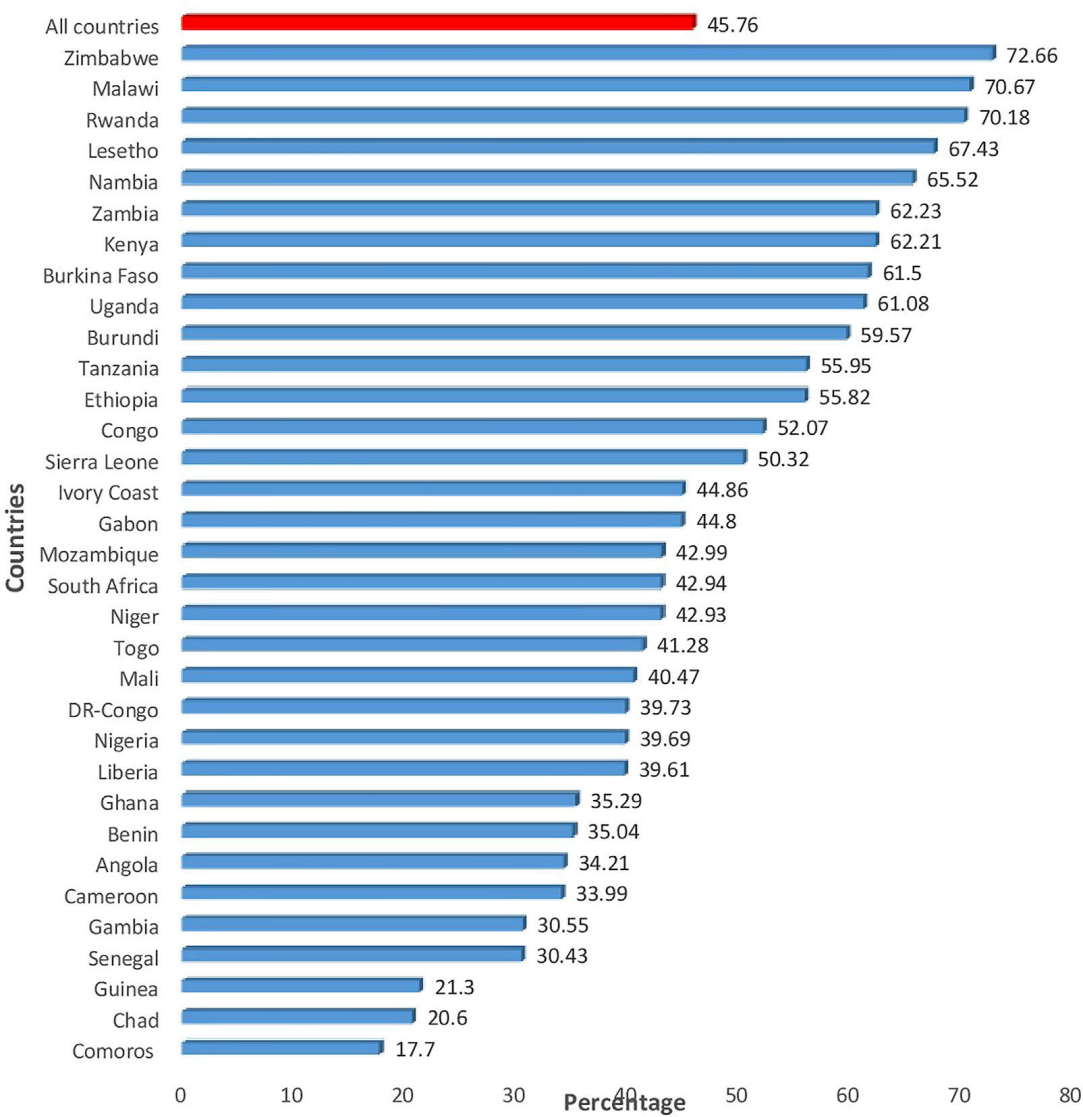
Intention to use contraceptive in countries in sub-Saharan Africa, 2010–2019.

A total weighted sample of 334386 women were included in the analysis. The median age of the study participants was 26 years (IQR: 19–35) and 61.5% of the women were rural dwellers. The majority (35.42%) of the women were educated to the level of secondary school or higher. Of the study participants 62% were in work and 66.71% had media exposure (Table 2). The majority (77.26%) of the women had four or above children. Half (50.27%) of the women were married at the age of 18 or older (Table 3). Overall, 45.76% (95% CI: 45.59, 45.93) of the women had intention to use contraceptives. From the 33 countries, Zimbabwe and Comoros accounted for the highest (72.66%) and the lowest (17.7%) intention to use contraception, respectively (Figure 1).

**TABLE 2:** Socio demographic and economic characteristics of the participants in countries in sub-Saharan Africa, 2010–2019.

Variables	Category	Frequency	Percent
Age in years	15–24	145555	43.53
	25–34	97571	29.18
	35+	91260	27.19
Residence	Urban	128743	38.50
	Rural	205643	61.50
Educational status of respondents	No education	118001	35.29
	Primary education	97947	29.29
	Secondary education	118438	35.42
	Higher education		
Husband education	No formal	90665	42.95
	Primary	51160	24.24
	Secondary and higher	69245	32.81
Occupation of respondents	Not working	121314	37.93
	Working	198485	62.07
Wealth	Poor	63455	18.98
	Middle	128800	38.52
	Rich	142131	42.50
Mass media exposure	Yes	222749	66.71
	No	111167	33.29
Distance to the health facility	Big problem	123597	38.43
	Not big problem	198055	61.57

**TABLE 3:** Obstetrics-related characteristics of mothers in countries in sub-Saharan Africa, 2010–2019.

Variables	Categories	Frequency	Percent
Ideal number of children	<4	76049	22.74
	≥4	258337	77.26
Age at first cohabitation	<18	114127	49.73
	≥18	115356	50.27
Number of living children	No	112307	33.59
	1–2	91498	27.36
	≥3	130581	39.05
Ever had terminated pregnancy	Yes	41757	12.49
	No	292580	87.51

Within clusters, there were significant differences in the intention of women to use contraceptives. In the baseline model without any determinant variables, 2.52% of the variance in the intention to use contraceptives could be explained by between-cluster variation of the characteristics (ICC = 0.0252). The between-cluster variation was decreased to 2.37% in the final model 3, which had both the individual and community level factors. According to this, the variation in the likelihood of intention to use contraceptives could be attributed to differences in clusters. In terms of goodness of fit, model 3, which incorporated both the individual and the community-level factors, was selected to predict the intentions to use contraceptives among reproductive age women. This model was selected because it has the lowest (391417.18) deviance as compared with the rest of the models**.**

After adjusting for the individual and community level variables, the final model is the complete model that shows the determinants of individual and community-level factors of intention to use contraceptives among reproductive-age women. Age of the women, education status of women, occupation, wealth index, number of living children, ideal number of children, media exposure, distance to the health facility, and residence were determinants of intention to use contraceptives among reproductive age women. Accordingly, intention to use contraceptives among reproductive aged women aged 15–24 years (*p* < 0.001) and aged 25–34 (*p* < 0.001) were 4.35 and 3.12 times higher than those women aged 35–49 years, respectively. Intention to use contraceptives among women who completed primary or secondary school and above were 67% (*p* < 0.001) and 88% (*p* < 0.001) times higher than those who did not have any education at all, respectively. Intention to use contraceptives was 22% higher among women who were in work than their counterparts (*p* < 0.002). Intention to receive contraceptives among women in the middle and rich wealth statuses were 10% (*p* < 0.001) and 21% (*p* < 0.001) higher as compared to the poor wealth categories, respectively.

Those women who had media exposure were 35% (*p* < 0.001) higher to intend to use contraceptives than women who had no media exposure. The likelihood of intention to use contraceptives among women who had 1-2 children, or with 3 or more children were 1.27 (*p* < 0.001) and 67% (*p* < 0.001) higher than women who had no children at all, respectively. The likelihood of intention to use modern contraceptives was 51% (*p* = 0.031) higher among women who had less than four ideal number of children than their counterparts. Among women who perceived distance to the health facilities as not a big problem had 8% (*p* < 0.002) higher likelihood to have the intention to use contraceptives compared with those who perceived distance to the health facilities as a big problem. The likelihood of intention to use contraceptive was 38% (*p* < 0.002) higher among rural resident women as compared with urban resident women (Table 4).

**TABLE 4:** Multilevel logistic regression models for individual and community determinants of intention to use contraceptives in countries in sub-Saharan Africa, 2010–2019.

Variables	Intention to use contraceptive		Model 1 AOR	Model 2 AOR	Model 3 AOR
	Yes n (%)	No n (%)	(95% CI)	(95% CI)	(95% CI)
Individual level factors					
Age in years					
15–24	80154 (55.07)	65401 (44.93)	4.32 (4.21, 4.43)		**4.35(4.23, 4.46)**
25–34	48985 (50.20)	48586 (49.80)	3.08 (3.02, 3.15)		**3.12(3.05, 3.19)**
35–49	23883 (26.17)	67377 (73.83)	1		1
Mother’s education					
No formal education	38807 (32.89)	79194 (67.11)	1		1
Primary education	49176 (50.21)	48772 (49.79)	1.70 (1.67, 1.73)		**1.67(1.63, 1.70)**
Secondary and higher	65039 (54.91)	53398 (45.09)	1.83 (1.79, 1.87)		**1.88(1.84, 1.92)**
Occupation			1		**1**
Not Working	55988 (46.15)	65327 (53.85)	1		**1**
Working	90523 (45.61)	107962 (54.39)	1.27 (1.25, 1.29)		**1.22(1.20, 1.24)**
Wealth index					
Poor	25268 (39.82)	38187 (60.18)	1		1
Middle	57460 (44.61)	71341 (55.39)	1.05 (1.03, 1.07)		**1.10(1.07, 1.12)**
Rich	70294 (49.46)	71837 (50.54)	1.02 (0.099, 1.04)		**1.21(1.18, 1.24)**
Media exposure					
Yes	110852 (49.77)	111897 (50.23)	1.39 (1.36, 1.41)		**1.35(1.33, 1.37)**
No	42019 (37.8)	69148 (62.20)	1		**1**
Number of living children					
No	59229 (52.74)	53078 (47.26)	1		**1**
1–2	44710 (48.86)	46388 (51.14)	1.25 (1.23, 1.28)		**1.27( 1.25, 1.30)**
≥3	49083 (37.59)	81498 (62.41)	1.65 (1.61, 1.70)		**1.67( 1.62, 1.71)**
Ideal number of children					
<4	44009 (57.87)	32041 (42.13)	1.53 (1.50, 1.56)		**1.51(1.48, 1.54)**
≥4	109013 (42.20)	149324 (57.80)	1		**1**
Community media exposure					
Low	77315 (45.23)	93612 (54.77)		1	1
High	75707 (46.32)	87752 (53.68)		1.03 (0.99, 1.08)	0.95 ( 0.91, 1.02)
Community education					
Low	79128 (44.69)	97936 (55.31)		1	1
High	73894 (46.97)	83429 (53.03)		1.11 (1.06, 1.15)	1.01 ( 0.96, 1.05)
Distance to the health facility					
Big problem	54194 (43.85)	69403 (56.15)		1	**1**
Not Big problem	95734 (48.34)	102321 (51.66)		1.19 (1.17, 1.20)	**1.08(1.06, 1.09)**
Residency					
Rural	92354 (44.91)	113289 (55.09)		0.99 (0.97, 1.01)	**1.38(1.35, 1.41)**
Urban	60668 (47.12)	68076 (52.88)		1	1

Random effect	Null	Model 1	Model 2	Model 3
ICC (%)	2.52	2.47	2.44	2.37
Variance	0.085	0.083	0.082	0.078
MOR	1.32	2.38	1.31	1.30
PCV (%)	Ref	2.35	3.53	8.24
Model fitness				
Deviance (-2LL)	462636.1	407011.26	441444.84	391417.18

Statistically significant at *p*-value < 0.05, AOR, adjusted odds ratio; COR, crude odds ratio, Null model: adjusted for individual-level characteristics, Model 2: adjusted for community-level characteristics, Model 3: adjusted for both individual and community-level characteristics.

## Discussion

The study attempts to assess the magnitude and associated factors of the intention to use contraceptives among reproductive age women in SSA. This study found that 45.76% (95% CI: 45.59, 45.93) of reproductive age women had the intention to use contraceptives, with Angola having the lowest intention of 20.30%. This finding is in line with a study done in Mozambique (44.7%) [21], but lower than studies conducted in Ghana 49.3% [22], 84.3% in Ethiopia [23], and 91% in the USA [24]. However, the results are higher than a study done in Pakistan 42.00% [25]. The discrepancy could be explained by the comprehensive nature of our study, the difference in the context, sample size, socio-demographic factors, access to information, and the availability of services used.

This study identified important factors that were associated with intention to use contraceptives. Age of the women, education, occupation, wealth index, media exposure, number of living children, and ideal number of children were the individual level factors, whereas distance to the health facilities and residency were community level factors.

In this study, respondents who were 15–24 and 25–34 years old were more likely to have the intention to use contraceptives than older age groups. It is consistent with the studies conducted in Ethiopia [19], Jordan [26], and Malawi [13]. One possible explanation might be that women in the 15–24 and 25–34 age groups are at a time at which most women engage in different activities to fulfill their needs. As a result, they want to postpone childbirth. This implies that they might have more intention to use contraceptives [27]. Moreover, there is a low risk of conception as a woman’s age increases [26].

Women with a formal education have higher odds of intention to use contraceptives in the future compared to those without formal education. The findings of this study are in agreement with studies conducted in Ethiopia [19] and Uganda [28]. One possible reason might be that women with a formal education have better exposure to contraceptives through the media, which improves their awareness and access to contraceptive alternatives and helps them to understand the health benefits of the contraceptive in reducing fertility, unintended pregnancy, unsafe abortions, and other maternal and child problems [29, 30]. In addition, educated women have greater autonomy in decision-making regarding contraceptive use [29]. This suggests that educating women will be one way to improve the intention of contraception use in these countries.

The likelihood of intention to use contraceptives among women from households in the middle and rich wealth quintiles was higher than that of women from poor households. This finding is supported by a study done in Mozambique [31]. One reason might be that women from rich households might be able to deal with the cost barrier associated with access to contraceptive use as compared to those from poor households, since they can overcome both the direct and indirect costs associated with contraceptive uptake [32]. Another possible reason could be that, due to an income increase, exposure to different types of information and financial accessibility of services will be improved [33]. Similarly, married women who had a job had higher odds of intention to use contraceptives than those who had not. This might be due to women who were self-employed being more educated, having better decision-making confidence, autonomy, easily accessible contraceptive methods, and even better living standards than those who had no job [34].

In this study, women who had media exposure had higher odds of intention to use contraceptives compared to their counterparts. This finding is similar to a study done in Jordan [26]. One reason for this may be that women who had media exposure might have a better understanding of contraception, which can bring a positive change in their attitude toward contraception and have a substantial positive effect on contraceptive use and intended future use of contraception [35]. The study indicated that media exposure would reduce the barriers to access and use of health care services, including future intentions of contraception.

Furthermore, women who perceive the distance to the health facilities as not a big problem were more likely to have the intention to use contraceptives. One possible explanation could be that women who perceive the distance to the health facilities as not a big problem have good awareness of contraception since they are more likely to receive counseling on family planning and receive the recommended maternal health care services [36]. In addition, previous reports found that distance to health care facilities is an important deterrent for women seeking health care services [37]. This result shows that improving geographical access to health care facilities increases the intention of contraceptive use.

Another important factor that significantly influenced the intention of contraceptive use in this study was ideal number of children. Women who have a small ideal number of children had their future intention to use contraceptives increased. This could be because women who have a small ideal number of children could be more educated, living in an areas where use of family planning is been supported, a positive attitude of family planning, and they achieved their desired number of children early [38].

Studies also found that those individuals residing in rural areas were more likely to have intention to use contraceptives as compared with those residing in urban areas. This is a seemingly counter-intuitive finding. Further studies are essential to unearth the reasons why married women who live in urban areas have low intention to use contraceptives.

The study’s main strength was that it used nationally representative DHS from 33 SSA countries, and therefore findings from the sub-region could be generalized. In addition, the DHS uses validated instruments in its appraisals of datasets along with its large sample size and well-designed procedures, such as training field enumerators and employing well-tested methods for data collection. Even if important findings were found in the current study, the cross-sectional nature of the study did not show the cause-and-effect relationship between the outcome and the explanatory variables. Since DHS data did not include qualitative data, we are unable to address the association of qualitative variables, such as socio-cultural factors, on the intention of contraception use.

In conclusion, intention to use contraceptives among reproductive age women in SSA remains low and this can result in low contraceptive prevalence and high rates of unintended pregnancies. Age of the women, women’s education, occupation, wealth index, media exposure, number of living children, ideal number of children, distance to the health facilities, and residency were significantly associated with intention to use contraceptives. Therefore, governmental and non-governmental organization in the various countries considered in the current study should put in measures that will halt barriers to intention to use contraceptives whilst intensifying mass education on intention to use contraceptives. This education should be more centered on women who are in socio-economically disadvantaged communities, those with high parity and high fertility preferences, and those who are not in work. Further studies are essential to unearth the reasons why reproductive age women who live in urban areas have low intention to use contraceptives.

## Data Availability

Data for this study were sourced from Demographic and Health surveys (DHS), which is freely available online at (https://dhsprogram.com).
